# Evidence-based capacity of natural cytochrome enzyme inhibitors to increase the effectivity of antineoplastic drugs

**DOI:** 10.1007/s12672-022-00605-y

**Published:** 2022-12-26

**Authors:** Laxmi Manthalkar, Ajazuddin, Sankha Bhattacharya

**Affiliations:** 1 Department of Pharmaceutics, School of Pharmacy & Technology Management, SVKM’S NMIMS Deemed-to-be University, Shirpur, 425405 Maharashtra India; 2 Department of Pharmaceutics, Rungta College of Pharmaceutical Sciences & Research, Khoka-Kurud Road, Bhilai, 490024 Chhattisgarh India

**Keywords:** Cytochrome enzymes, CYP450, Natural CYP inhibitors, Biotransformation, Cancer, Targeted prevention, Primary care, Secondary care, Thymoquinone, Quercetin, Garlic

## Abstract

**Abstract:**

Cytochrome (CYP) enzymes catalyze the metabolism of numerous exogenous and endogenous substrates in cancer therapy leading to significant drug interactions due to their metabolizing effect. CYP enzymes play an important role in the metabolism of essential anticancer medications. They are shown to be overexpressed in tumor cells at numerous locations in the body. This overexpression could be a result of lifestyle factors, presence of hereditary variants of CYP (Bio individuality) and multi-drug resistance. This finding has sparked an interest in using CYP inhibitors to lower their metabolizing activity as a result facilitating anti-cancer medications to have a therapeutic impact. As a result of the cytotoxic nature of synthetic enzyme inhibitors and the increased prevalence of herbal medication, natural CYP inhibitors have been identified as an excellent way to inhibit overexpression sighting their tendency to show less cytotoxicity, lesser adverse drug reactions and enhanced bioavailability. Nonetheless, their effect of lowering the hindrance caused in chemotherapy due to CYP enzymes remains unexploited to its fullest. It has been observed that there is a substantial decrease in first pass metabolism and increase in intestinal absorption of chemotherapeutic drugs like paclitaxel when administered along with flavonoids which help suppress certain specific cytochrome enzymes which play a role in paclitaxel metabolism. This review elaborates on the role and scope of phytochemicals in primary, secondary and tertiary care and how targeted prevention of cancer could be a breakthrough in the field of chemotherapy and oncology. This opens up a whole new area of research for delivery of these natural inhibitors along with anticancer drugs with the help of liposomes, micelles, nanoparticles, the usage of liquid biopsy analysis, artificial intelligence in medicine, risk assessment tools, multi-omics and multi-parametric analysis. Further, the site of action, mechanisms, metabolites involved, experimental models, doses and observations of two natural compounds, quercetin & thymoquinone, and two plant extracts, liquorice & garlic on CYP enzymes have been summarized.

**Graphical Abstract:**

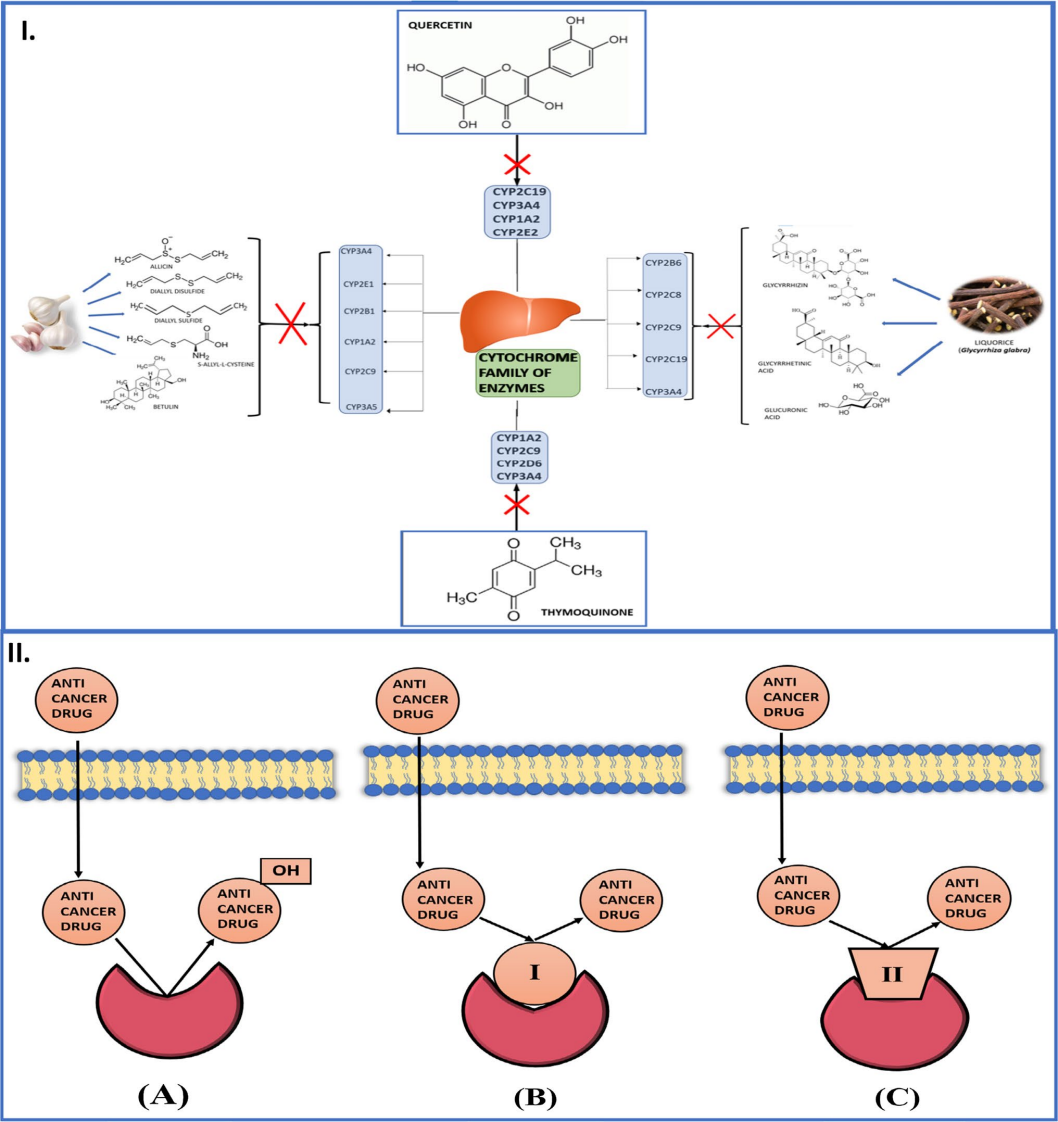

## Introduction

**Fig. 1:**
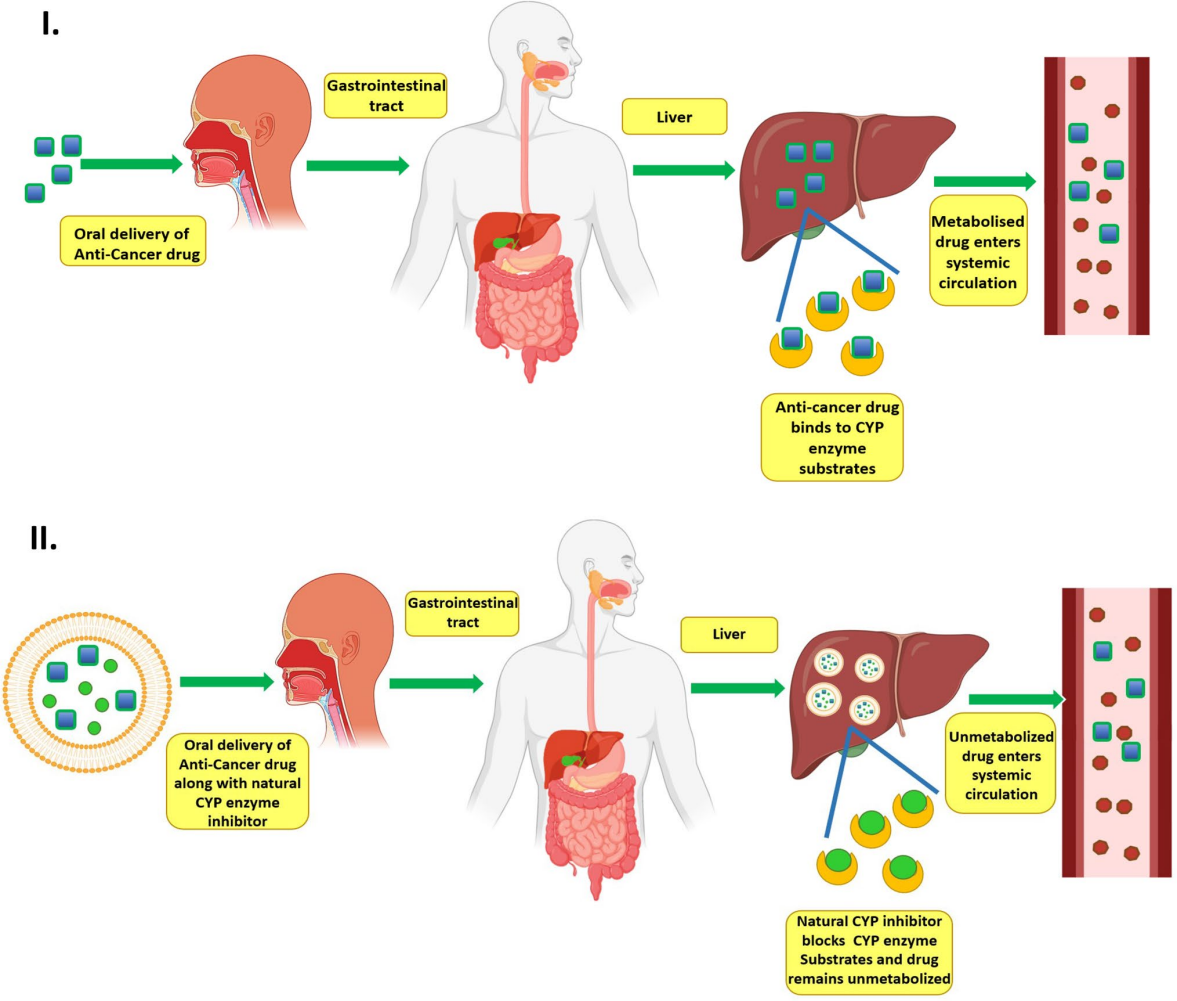
**I** Fate of the anti-cancer drug in the body in the absence of a natural cytochrome inhibitor when administered on its own leads to biotransformation of the drug. **II** Fate of the drug when administered in the form of a novel drug delivery system along with the natural CYP inhibitor inhibitor leads to better systemic absorption of the drug in its unchanged form

One of the most important methods for treating malignancies is chemotherapy. Chemotherapeutic medications are used to slow the development and size of tumors, however for the majority of these treatments, the ability to slow tumor growth eventually declines due to diverse resistance mechanisms [1]. One of the key contributors to the development of resistance to anti-cancer medications has been discovered as the overexpression of human cytochrome enzymes [2]. Steroids, fatty acids, eicosanoids, vitamins, xenobiotics, and some other unknown substrates are among the principal substrates of human CYP450s, from which CYP450s can be loosely divided into six categories. A vast range of bioactive compounds, including steroid hormones, polyunsaturated fatty acids, eicosanoids and fat-soluble vitamins, are bio-transformed and catabolized by CYP450s. Additionally, it is recognized that some CYP450s are the key enzymes in phase I drug metabolism, which is crucial for the biotransformation and removal of drugs and toxins. Typically, following an oxidation reaction catalyzed by CYP450, drugs are changed to a less potent form and then move through the metabolizing process. In contrast, CYP450s are necessary for the creation of some medicines' pharmacologically active forms in order to achieve optimal pharmacokinetics, prevent toxicity, and enhance targetability. It has been demonstrated that genetic changes in the CYP450 genes are associated with a high risk of developing various types of malignant tumours and can affect how well CYP450-metabolizing anticancer medicines work therapeutically [3]. CYP450 is a family of Heme enzymes that are monooxygenases and have heme as their cofactor [4]. They are located mainly in the liver and said to be responsible in phase—1 reactions and metabolism of more than 80% drugs [5]. These cytochrome enzymes are a category of hemoprotein monooxygenases that catalyze the oxidation of several xenobiotic and endogenous substances [6]. CYP enzymes are also known to regulate various other drugs like antifungals, Fluconazole and other azole antifungals block the activity of the cytochrome P450-dependent 14-alpha-sterol demethylase [7]. Studies have shown that this resistance can be overcome by using Cytochrome inhibitors in conjunction with chemotherapeutic drugs [8]. This technique of blocking a certain enzyme/substrate gene expression in order to achieve a desired therapeutic result has long been used in other diseases too other than cancer. Like for example, by specifically blocking *P. falciparum*’s electron-transport chain at the cytochrome bc1 level, 4(1H)-pyridones work against malaria (complex III) [9]. Similarly inhibition of histone acetyltransferases KAT6A/B have been helpful in arresting tumor growth [10]. CYP enzymes specifically CYP1B1 have been found to be very highly expressed in human cancer cells of the breast, colon, lung, esophagus, skin, lymph node, brain, and testicles, but negligibly in healthy tissues [11]. CYP enzymes are also known to catalyze the activation of procarcinogens-1 CYP1B1 is specifically known for the hydroxylation of compounds [12]. In addition to catalyzing the 4-hydroxylation of estrogens, which is thought to be a crucial step in hormonal carcinogenesis, CYP1B1 also activates an array of environmental mutagens [13]. In a study by Guengerich Et. Al. CYP enzymes specifically CYP1B1 was found to be significant because: (1) P450 1B1 has been shown to activate a wide range of procarcinogens; and (2) P450 1B1 appears to be the main catalyst in the 4-hydroxylation of estrogens (at least in extrahepatic tissues): (i) Due to the prevalence of cancers, P450 1B1 is expressed in a variety of interesting tissues; (ii) 4-hydroxylation has been linked to increased estrogen carcinogenicity, though it is unclear whether this is due to increased estrogen reactivity with DNA or the production of reactive oxygen species; and (iii) research using animals deficient in P450 1B1 demonstrates how important this enzyme is for the development of tumors brought on by 7,12-dimethylbenz[a]anthracene [14]. It was also discovered that hereditary variants in CYP3A4 are a notable hotspot for patient-to-patient variation in plasma levels, negative side effects, and pharmacological responsiveness to drugs. The largest member of the CYP3A subfamily is the human cytochrome P450 3A4 (CYP3A4), which accounts for between 30 and 60% of all CYP450s in adult liver. According to a docking study by Khan et al., rationally synthesizing natural analogues in relation to synthetic pharmaceuticals might result in medications with enhanced therapeutic benefits for chemoprevention. A lot of different medications are metabolized by CYP3A4, and we can regulate the selective drug metabolism by inhibiting CYP3A4 with the aid of flavonoids [15]. Ketoconazole, a CYP3A4 inhibitor, or rifampicin, a CYP3A inducer, can be used in clinical research to measure the alteration of drug exposure before and after the inducer/inhibitor. This can help predict drug-drug interactions, such as those involving CYP3A4 for example. Less research has been done on the inhibition or induction of other CYPs, though [16]. Another reason for inactivity of anti-cancer drugs is the development of Multidrug resistance. It has been seen that CYP enzymes are involved in the encoding of genes for various diseases including cancer and *Mycobacterium tuberculosis*. To overcome this, many researchers are going deep into the physiology, biochemistry & molecular interventions which are disease specific [17]. Although our area of concern is regulating the overexpression of CYP enzymes, the metabolizing action of cytochrome enzymes is not always undesirable. They play a key role in converting the prodrug into the therapeutically active form of the drug. Thus, the cytochrome P450 system's role in prodrug activation offers some guidance on prodrug design that is especially adaptive for directing drug activation to the liver, malignancies, or hypoxic tissues [18]. Hence, from all the literature review done for this article, alteration, biotransformation, degradation and eventually reduced therapeutic activity of antineoplastic drugs can be attributed to the following four traits of CYP enzymes—(i) Overexpression of CYP enzymes; (ii) Hereditary variants of CYP (Bio individuality); and (iii) Development of Multidrug resistance. Flavonoids are one of the most widely known natural cytochrome inhibitors, one of which is thymoquinone. They are secondary plant metabolites that can be found in foods including fruits, vegetables, tea, wine, propolis, and medicinal plants. These substances are of interest because of their biological features that have an impact on human health and help organoleptic aspects of meals (such as the color and flavor of tea and wine) [19, 20]. Flavonoids have beneficial pharmacological effects, such as antitumor action. They are the primary plant pigments that function as cell cycle inhibitors, physiological regulators, and chemical messengers. One of the most well-known and widely used compounds from plant sources is flavonoids. Natural goods, vegetables, leguminous plants, and even some types of greens contain them. The C6-C3-C6 framework 1-benzopyran makes up the skeleton of flavonoids [21]. It has been demonstrated that certain flavonoids can suppress the activity of the cytochrome P450 enzymes in a research by Konda et al. The results of the study demonstrated that paclitaxel was more orally bioavailable when combined with flavonoids. This was primarily due to increased absorption in the gastrointestinal tract due to P-glycoprotein inhibition and decreased first-pass metabolism of paclitaxel due to flavonoid inhibition of the CYP3A subfamily in the small intestine and/or liver. As a consequence, the in vivo inhibitory potential of flavonoids was confirmed [22]. A flavonoid called quercetin is present in substantial concentrations as its glycosides and aglycone in a number of plants and dietary supplements. Due to quercetin's high pre-systemic biotransformation rate, its conjugates are primarily what are found in circulation. Previous investigations have shown that quercetin can interact with a number of proteins that are significant for pharmacokinetics. However, very little study has been conducted on the interactions of its metabolites with drug transporters and enzymes involved in biotransformation [4, 23]. Similarly the black seeds of the Nigella sativa (NS) plant contain a significant amount of thymoquinone (TQ), a bioactive chemical which is known to inhibit cytochrome enzymes. It has been observed that Thymoquinone substantially lowered the levels of the expression of CYP3A4, CYP3A7, and CYP17A1 in treated cells [24]. Plant extracts are isolated forms of the herbal component. Extracts may comprise of multiple different compounds. The intent behind selection of these four natural inhibitors is that their potential to alter CYP expression during chemotherapy has not been exploited yet to their fullest potential and these are the most commonly used plant-derived cytochrome inhibitors. This is essential and an area that needs to be explored because over expression of these enzymes leads to metabolism and degradation of antineoplastic drugs leading to a substantial decrease in their therapeutic activity. Hence, to increase the therapeutic activity, overcome multi drug resistance, decrease dosage and thus decrease the cytotoxic load caused in the cells due to antineoplastic drugs, cytochrome enzymes like quercetin, thymoquinone, garlic extract and liquorice extract could be used in order to inhibit cytochrome activity, so that antineoplastic drugs show optimum therapeutic activity. A diagrammatic representation of the fate of the drug in presence and absence of the natural CYP enzyme inhibitor is depicted in Fig. 1. In addition to that it has also been seen that flavonoids are able to prevent the formation and proliferation of tumors and cancer cells through a variety of molecular processes. Over the last 10 years, cancer prevention has paid particular attention to their effects on procarcinogen-activating enzymes, particularly the cytochrome P450 family.

## Natural substances in cancer management, primary, secondary and tertiary care

Despite the fact that a number of inhibitors for CYPs genes are recognized, the main disadvantage of other synthetic inhibitors is that they contain the steroid scaffold, which leads to the unfavorable side effects (dyspnea, edema, contusion, etc.) seen in patients. In this context, there has been a continuous search for non-steroidal scaffolds such as natural compounds that will inhibit CYP enzymes more selectively and effectively [25]. Often times in alternative system of medicine extracts of herbs are given directly. The present review focuses on discussing the potential of quercetin, thymoquinone, garlic extract and liquorice extract in effective inhibition of cytochrome enzymes that are overexpressed in cases of tumor growth. Out of the four topics garlic and liquorice stand apart from thymoquinone and quercetin in the sense that they are extracts and not compounds. According to our current knowledge, particularly in targeted and combination therapy [26], nanotechnology can improve the bioavailability and accessibility of hydrophobic substances and be employed as drug delivery systems. Phytochemicals in humans exhibit limited bioavailability and absorption, quick elimination, resistance, and toxicity. Despite being in the early stages of clinical studies, the decrease of the aforementioned drawbacks by using its nanoparticles has enormous promise for the treatment of cancer in the future. The stability and resistance to enzymatic activity in the digestive tract are increased by the use of small particle sizes and distinctive materials in delivery methods [27]. Chronic inflammation happens to be one of the root causes for cancer initiation. By controlling immune cells, reducing pro-inflammatory chemokines, cytokines, and COX-2, and inhibiting PI3K/AKT and IKK/JNK, flavonoids have anti-inflammatory effects. Flavonoids play a major role in all three stages of cancer namely cancer initiation, promotion and progression [26, 28]. In the framework of 3P medicine, protective anti-cancer applications of flavonoids should adhere to the principles of individual prediction, targeted prevention, and personalized therapy algorithms. In order to do this, it is highly advised to use specialist analytical methods to a companion diagnostic, such as liquid biopsy analysis, risk assessment tools, multi-omics and multi-parametric analysis, and the use of artificial intelligence in medicine [29]. Flavonoids are thought to be powerful radiosensitizing chemicals for cancer cells, despite having radioprotective effects on healthy cells. By preventing methylation of the Keap1 gene promoter area, genistein selectively reduced radio sensitizing effects in non-small cell lung cancer (NSCLC) A549 cells; hypermethylation of the Keap1 promoter resulted in chemo/radio resistance mediated by the Nrf2-Keap1 pathway [30]. According to Hou et al. [28], flavonoid treatment prevents recurrent colitis and colitis-related tumorigenesis. These flavonoids also prevent inflammation-induced colorectal cancer in vitro by downregulating IL-1 and TNF-.

## Targeted prevention of cancer on cytochrome enzymes

A number of cellular and molecular pathways are involved in the complex disease of cancer. One of the leading causes of death worldwide is cancer [31]. A universal metabolic network revealed a metabolic route that was found to bioactivate chemicals and induce cancer. This network included phase I xenobiotic enzymes, CYPs, and phase II xenobiotic-conjugating enzymes. Numerous studies have discovered that P450s (1A1, 1A2, 1B1, 2A6, 2A13, 2E1, and 3A4) are involved in the activation of a variety of environmental carcinogens, such as tobacco-related nitrosamines and polycyclic aromatic hydrocarbons (PAHs) [32–34]. The most significant enzymes that catalyse antineoplastic agent-related reactions are P450s. Cytotoxic antineoplastic drugs are still a crucial component of chemotherapy in the treatment of malignant cancers. The metabolism of anticancer medications is mostly controlled by the CYP2A, CYP2B, CYP2C, CYP2D, and CYP3A subfamilies. Chemoresistance is one of the most significant issues in the treatment of brain malignancies [35, 36]. Intracellular drug inactivation may be caused by elevated P450 concentrations. Additionally, because P450s expressed in tumours may be implicated in the activation and/or inactivation of chemotherapeutic drugs, it has been proposed that local expression of P450s in tumours is crucial for the management of cancer. It was also observed that in Prostate Cancer Therapy phytochemicals help in minimizing the adverse effects of chemo- and radio-therapy [37] Hence by targeting these specific mechanisms and routes via which tumor development takes place, cancer can be mitigated and treated.

### Anti-cancer effects and effect of phytochemicals: primary, secondary, and tertiary care

The targeted prevention of cancer initiation is possible because plant bioactive substances have special capabilities. Despite the fact that phytochemicals' Geno protective effects are well known, there aren't any publications that systematically outline the range of anticancer effects of phytochemicals, plant extracts, and diets derived from plants that are relevant to stratified patient groups at the level of targeted primary (cancer development) and secondary (cancer progression and metastatic disease) prevention [38]. In a study conducted by Fatemah et al. the distinct expression profile of CYP1B1 in cervical cancer tissues compared to normal cervical tissues raises the possibility that CYP1B1 could be exploited as a therapeutic target in the future. Although it was not expressed in samples of normal, healthy cervical tissue, the majority of cervical cancer samples (91/100, 91.0%) did. The expression of CYP1B1 varied significantly (P = 0.01) between tumorous and benign cervical tissues. Additionally, it was discovered that the frequency of CYP1B1 expression was considerably greater in patients with advanced disease grades (P = 0.03) and in patients who had lymph node metastases (P = 0.01). Surprisingly, patients with a high incidence of the human papilloma virus 16/18 had considerably increased CYP1B1 expression (P = 0.04) [39].

## Mechanism by which anti-cancer drugs are metabolized

**Fig. 2:**
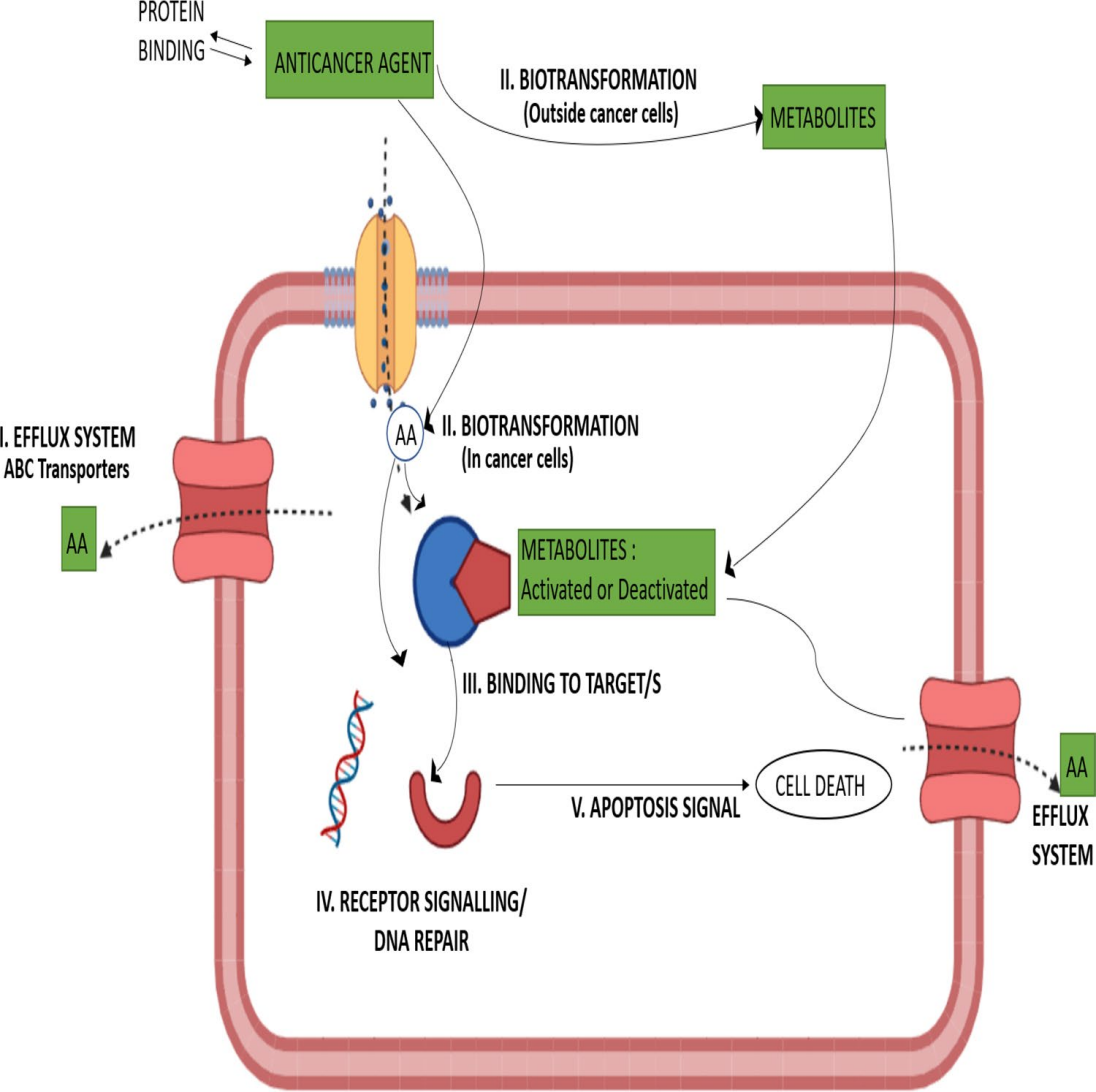
The body or cancer cells (a) may develop and exhibit key anticancer drug (AA) resistance mechanisms. They reduce amounts of active compounds or medicinal effectiveness. Anticancer drugs may undergo biotransformation in the liver or in the tumor cells as a cause of resistance. **I** Adenosine triphosphate (ATP)-binding cassette (ABC) transporter activity is part of the efflux system (e.g., ABCB 1, also known as P-glycoprotein). **II** Drug inactivation or prodrug activation through biotransformation is accomplished by metabolizing enzyme activity (e.g., cytochrome P450450 [CYP]). **III** Target binding: enhanced DNA repair, mutation, or overexpression of the target protein. **IV** Modulation of co-regulator proteins (co-activators activators or) to change receptor signaling co-repressors). Desensitization of apoptotic signals in **V** programmed cell death (apoptosis)

Cytochrome enzymes catalyze various oxidation and reduction reactions. In the literature, around 95% of these are carried out by cytochrome P450 (CYP) enzymes. The majority of CYP450 processes are oxidations and utilize molecular oxygen, or O_2_ [40]. CYP450s nearly generally work as monooxygenases or mixed-function oxidases with NADH or NADPH acting as a cofactor for the pyridine nucleotide (used to deliver electrons via a flavoprotein, sometimes via an iron-sulfur protein) [41]. P450s also catalyze more intricate oxidations, such as the creation and breaking of C–C bonds, in addition to hydroxylation. Even more odd processes, such as cyclopropanation, can be catalyzed by enzymes developed from P450s by directed evolution [41]. In the catalytic cycle, on the side opposite to the axial thiolate, the substrate attaches close to the heme group. The active site's shape changes as a result of substrate interaction, frequently displacing a water molecule from the heme iron's distal axial coordination location [42]. Electron transfer from NAD(P)H is induced by substrate binding and is carried out by cytochrome P450 reductase or a different related reductase [43]. The resultant ferrous heme center is then bound by molecular oxygen at the distal axial coordination location, initially producing an oxy-myoglobin-like dioxygen adduct. A second electron is transported, perhaps from ferredoxins, cytochrome b5, or cytochrome P450 reductase, reducing the Fe-O_2_ compound to produce a transitory peroxo state. When the peroxo group created in step 4 is quickly protonated twice, one molecule of water is released, and P450 Compound 1, a highly reactive species, is created (or just Compound I) [44]. The fate of an anticancer agent inside the cell and in the extracellular medium is depicted in Fig. 2. The anticancer agent is either able to bind to the target site, show its action and destroy the tumor cell (cell apoptosis) or the drug might metabolize and get effluxed out without reaching the target site through the efflux system which is the ABC transporters in our case. It is seen that similar substances may co-induce the genes for CYPs and ATP-binding cassette B1 transporters, such as ABCB1, commonly known as P-glycoprotein [P-gp] or MDR1. Multiple findings highlight the overlap in the substrate selectivity of the CYP3A and ABCB1 transporters, even though no systematic link can be inferred. Cells have evolved the complimentary abilities to actively transport substances outside of the cell and to bio transform substances in order to defend against their possible hazardous effects over the course of billions of years (Fig. 1). Both methods, which may have co-evolved, may contribute to drug resistance by reducing the concentration of active medicines in the target cell and the systemic circulation [45]. The inhibition of the CYP enzymes could either be competitive or non-competitive (Fig. 2).

## Role of cytochrome P-450 in cancer

**Fig. 3:**
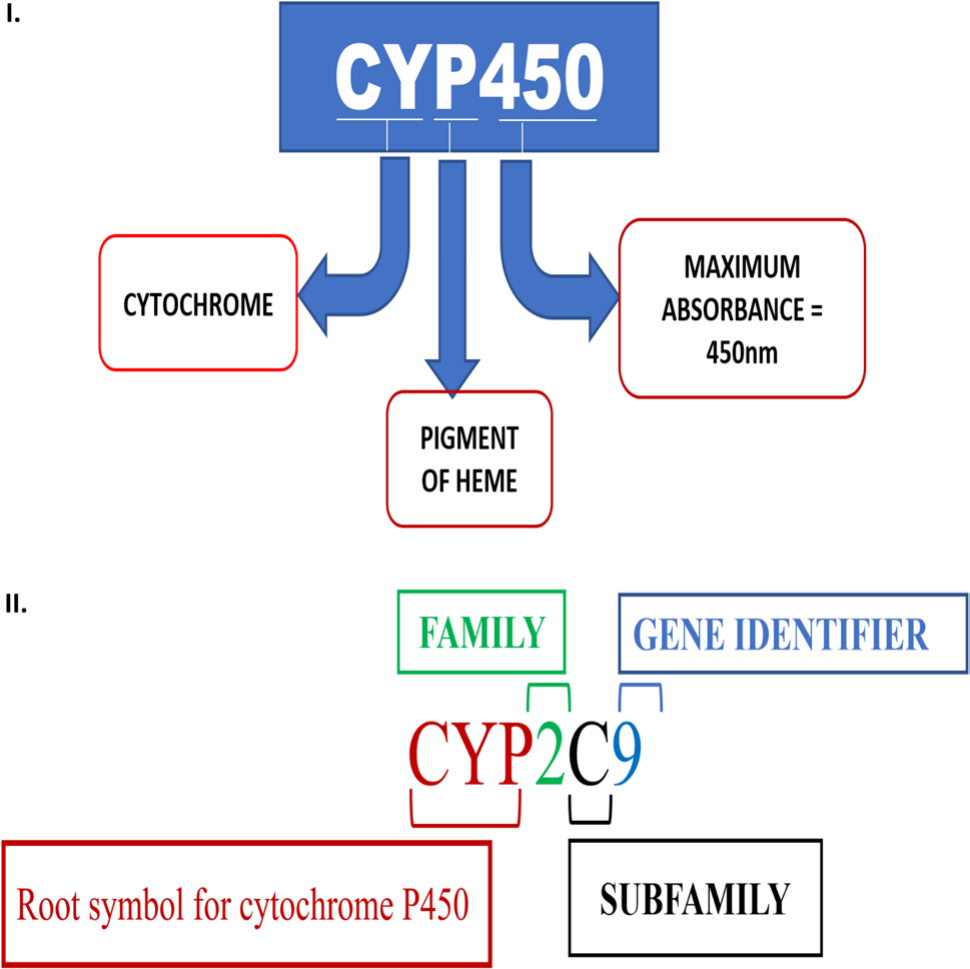
**I** Diagrammatic representation of what CYP450 stands for. **II** Nomenclature for cytochrome family of enzymes

The nomenclature of CYP enzymes is done by following a certain pattern which is depicted in Fig. 3. The biotransformation of xenobiotics depends on CYP enzymes, which are also linked to the growth of cancer and inflammation. The inflammatory process often exists both locally and systemically in the tumor microenvironment. Released cytokines, such as IL-1, IL-6, and TNF-, may then reduce the expression of CYP genes, particularly those from the CYP1, CYP2, CYP3, and CYP4 families. Changes in CYPs in cancer may affect how chemotherapeutic medications are metabolized in cancer patients, especially in polychemotherapy protocols where CYP enzymes may be suppressed [46]. CYP enzymes also play a crucial part in the metabolism of endogenous and exogenous substances. Additionally, they oversee turning procarcinogens into carcinogens. The primary CYPs involved in procarcinogen activation are CYP1A1, CYP1A2, CYP1B1, CYP2A6, and CYP2E1, whose gene polymorphisms are linked to an increased risk for certain malignancies [47]. Additionally, CYPs are linked to tumor growth and development, as well as the activation of prodrugs used to treat cancer and their metabolic clearance [48]. CYP2C9, CYP3A4, CYP1A2, CYP1A1, CYP2C9, CYP2D6, CYP2B1 and CYP2B2 are some enzymes that are major contributors of metabolism of drugs, hence it is important to identify the active sites, the structure, and the functional groups and ligands that interact with the drugs [49]. Cancer risk that involving CYP regulation cannot be predicted due to the complexity of carcinogen metabolism, because it is mediated by the activity of one or more genes [50]. Many pre-carcinogens are metabolized by the CYP family, producing secondary metabolites that have the ability to destroy DNA, create chemical adducts, and affect the mobilization and immobilization of anticancer medications [51]. About 1% of the human population exhibits stable polymorphisms or differences in gene sequences [52]. Polymorphic CYP enzymes are crucial for detoxication via drug metabolism, and they may be responsible for patient variation in the clinical course of different malignancies [53]. Purpose of a study conducted by LEE el et al. aimed at determining if CYP450 significantly affects breast cancer. Fifteen female mice were divided into control, exercise and drug treatment (Taxol 16 mg/kg). The human breast cancer model used was the MCF-7 human breast adenocarcinoma cell line. Between the exercise (0.75 ± 0.49 and 0.54 ± 0.50, respectively), pharmacological treatment (1.15 ± 0.38 and 1.55 ± 0.63), and control groups (1.32 ± 0.32 and 1.02 ± 0.23, P > 0.05) there was no difference in the transcript expression of CYP2E1 and CYP1A2. Similarly, there were no differences in the liver levels of CYP1A2 and CYP2E1 between the exercise and drug treatment groups (P > 0.05) (exercise: 55.84 ± 13.74 and 75.69 ± 16.62; drug treatment: 114.28 ± 19.20 and 151.95 ± 0.54; controls: 95.18 ± 18.13 and 98.17 ± 14.39). However, the exercise group had higher CYP2D6 expression (79.06 ± 28.03) than the controls (28.71 ± 6.63; P < 0.05) did. This establishes that breast cancer/ tumor growth is differentially affected by CYP450 [54]. In the report by Shahraki el et al. the investigation on was studied that may have an etiological title role in breast cancer. Generally, the objective of xenobiotic metabolism is to enhance the water solubility of numerous foreign chemicals to eradicate them from our physiological system; and this process happens in two phases. Cytochrome P450 enzymes are involved in phase I reaction, which sometimes transforms biologically inactive compounds into active or toxic metabolites. In this investigation, a comprehensive examination was performed into the expression of a wide range of cytochrome P450s in breast cancer. CYP11A1, CYP1A2, CYP19, CYP19A1, CYP21, CYP1A1, CYP1B1, CYP3A4, CYP3A5, CYP2D6 and CYP17 are all associated with breast cancer [55]. Tamoxifen, a drug widely used for the treatment of breast cancer selectively modulates estrogenic receptor. This drug TAM undergoes metabolism on interaction with the family of CYP450 enzymes that includes CYP2 enzymes namely CYP2D6, CYP3A5 and CYP2C19. However here the researcher studies the connection between the polymorphs of CYP450 with breast cancer. Thota et al. used genomic DNA and polymerase chain reaction restriction fragment length polymorphism (PCR–RFLP) analysis to determine whether or not the prevalence of the four most prevalent polymorphs in the CYP2D6*4 (G1934A), CYP2D6*10 (C188T), CYP3A5*3, and CYP2C19*2 alleles have any relationship to breast cancer [56]. CYP2D6 metabolizes Tamoxifen to endoxifen. Hence, we see a reduction in activity of the anticancer effect of tamoxifen due to the activity of CYP2D6 enzyme and other enzymes from the same family. The conclusion that was drawn stated clearly that polymorphs in CYP2 appear to have an association with breast cancer. The data of the conducted research along with the old reports together go on to establish that gene polymorphs of CYP enzymes prevalent in different populations may become a hinderance to tamoxifen therapy in breast cancer cases [57].

## Natural compounds as cytochrome inhibitors

### Thymoquinone

Thymoquinone (TQ), a bioactive, phytochemical substance is the main active constituent of Nigella sativa plant. It is non-toxic and has a wide range of therapeutic uses, including the treatment of cancer and diabetes. It is also used to reduce the intensity of diseases like Asthma, inflammation, arthritis, gastro, and liver problems. It has been labelled as a pan-assay interference chemical that binds to several proteins without preference [58]. TQ has also shown potential for usage in cancer-associated thrombosis as it has a very nominal effect on normal blood coagulation. In a study conducted by Wang et al. it was demonstrated that TQ competitively inhibited the conversion of warfarin into 7-hydroxywarfarin. CYP2C9 is an enzyme involved in the metabolism of warfarin. It was found that TQ is effective in inhibiting the activity of this enzyme [59]. It was seen that TQ exhibits an anti-genotoxic effect by optimizing the effect of CYP450 enzymes. The major activation of most polycyclic aromatic hydrocarbons (PAHs) including B(a)P, is mediated by CYP1A1/A2 class of cytochrome P450 isozymes. In a study by Badary Et. Al. TQ blocked the enzymatic activity of hepatic CYP1A1 when TQ was orally administered at a constant dose for a duration of 2 weeks. CYP1A1 is known to enhance oxidative stress, hence inhibition of this enzyme by TQ leads to minimal oxidative stress [60]. The research by Albassam et al. aimed at studying how thymoquinone (TQ) could affect the metabolic functioning of four drug metabolizing enzymes in human liver microsomes, namely P450 (CYP) 1A2, CYP2C9, CYP2D6 and CYP3A4. TQ, the key active constituent of *Nigella sativa* L. (Black Seed), a monoterpene is used as a cure for many ailments like headache, asthma, eczema, gastrointestinal problems, cancer, depression and hypertension across many different countries in traditional systems of medicine. *N. sativa* seed extracts possess inhibitory action towards cytochrome P450 3A4 AND CYP2D6 both in vivo and in vitro. It also leads in substantial reduction in gene expression and metabolic activity of CYP2D. Respective typical substrates were incubated with human liver microsomes (containing CYP enzymes) and NADPH with TQ at doses of 0, 1, 10 and 100 µM to evaluate TQ’s inhibitory effect on CYP enzymes (Table 1). Metabolites were formed as a result of interaction between the enzymes and substrates which were then analysed using HPLC. It was found that metabolic activity of all 4 selected enzymes were inhibited [24]. The results that were obtained from the research suggest a high possibility of drug interactions and instabilities in plasma concentration of a drug, leading to alterations in the pharmacological actions when the drug is administered with TQ or with herbs containing TQ. Percentage of metabolites produced at various TQ concentrations. The following substances were examined: [(A) acetaminophen (B) 4-hydroxytolbutamide (C) dextrorphan-d-tartrate (D) and (E) 6-b-hydroxytestosterone in pooled Human liver microsome (n = 3, mean SEM), *p < 05 compared to control]. TQ may change the metabolic expression of the CYP1A2, CYP2C9, CYP2D6, and CYP3A4 enzymes in humans since it is a very effective inhibitor of these enzymes [24]. A brief overview of the studies conducted to check the action of TQ on CYP enzymes is mentioned in Table 2.

**Table 1:** Percentage of Inhibition of expression of enzyme observed at different concentrations of TQ

Name of CYP enzyme	Name of metabolite formed	1 µM (%)	10 µM (%)	100 µM (%)
CYP1A2	Acetaminophen	24	14	82
CYP2C9	4-Hydroxytolbutamide	46	~ 70	~ 70
CYP2D6	Dextrorphan-d-tartarate	20	11	~̴̴̴28
CYP3A4	6-β-Hydroxytestesterone	~ 10	~ 24	79

**Table 2:** An overview of studies conducted to study the action of TQ on CYP enzymes

Drug	Experimental models	Dosages	Function/mechanism/observation	References
TQ	Human blood cells	1, 3, 10 and 100 μM	Repressed the conversion of exogenous arachidonic acid into 5-hydroxy eicosatetraenoic acid, which inhibited the synthesis of LTB4 and LTC4 and 5-LO function	[48]
*N. sativa* (ethanolic extract) TQ	Male Wistar rats	N. sativa (1000 mg/ml) and TQ (5–10 mg/ml)	Enhanced cytokine balance between Th1 and Th2 and NK cytotoxic activity	[49]
TQ	Human osteoarthritis chondrocytes	5–20 μM	Prevented the formation of inflammatory mediators by inhibiting the NF-B and MAPK signalling pathways	[50]
TQ	Rat	1 mg/kg	Decreased total leukocyte counts, total immunoglobulins (Igs), and antibody hemagglutination to protect against IC-induced immunotoxicity	[51]
TQ	T cells	0.52–10 μg/mL	Enhanced CD62L and CD8+ T cell expression, enhanced T cell survival	[52]
TQ	Cataracts	5 mg/kg	Through regulating the expression of Bcl-2, PD-1, Bax, and caspase-3, protected against gamma-irradiation-induced T cell fatigue and death	[53]
TQ	Human RA-FLS and Rat	0–10 μM 5 mg/kg	By preventing the activation of p38 mitogen-activated protein kinase, extracellular regulated kinases 1 and 2, and NF-kB-p65, the levels of IL-1, TNF, MMP-13, cox-2, and PGE(2) were reduced	[54]
TQ	Rat	10, 20, and 40 mg/kg/day	Formation and buildup of amyloid (A), as well as a decline in TNF- and IL-1	[48, 55]
TQ	Pancreatic ductal adenocarcinoma cells	75 μM	Stimulation of the genes for l-1, TNF, MCP-1, and COX-2	[56]
*N. sativa*’s oil	Peripheral blood mononuclear cells from humans	10 mg/kg	Prostaglandin E2 synthesis and stimulation of T-cell proliferation	[57, 58]

### Quercetin

Quercetin is a flavonoid commonly found in plants and widely marketed as a dietary supplement which is useful in cancer prevention [18, 19] and is said to have shown antiallergic, anti‐inflammatory, and antiviral activities [61]. It hinders the propagation of various types of cancers, such as lung, prostate, liver, breast, colon, and cervical [61]. Quercetin prevents cancer by various different mechanisms which involve cellular signaling, and inhibition of enzymes involved in expression of activity of tumor cells. It binds to cellular receptors and proteins [62, 63]. It was also reported that quercetin showed a synergistic effect when taken along with antineoplastic agents such as cisplatin, additionally improving the outcomes of the traditional chemotherapy (Brito et al. 2015). It has been seen that it inhibits cytochrome enzymes, including CYP1A1,1B1, 2C19, 2D6, 2C9, and 3A4 [64]. Losartan is a substrate for both CYP enzymes and P-gp, therefore altering CYP and P-gp activity may have a major impact on how losartan and its active metabolite EXP3174 are metabolized. By way of the cytochrome P450 enzymes CYP3A4 and CYP2C9, losartan may be swiftly absorbed after oral administration and converted into its active metabolite, EXP3174, which is around ten times more effective than the original medication. It was found in a study by Zhao et al. that when quercetin and losartan are delivered together to rats, there may be an herb-drug interaction. Quercetin may enhance losartan's systemic exposure while lowering the plasma levels of EXP3174, maybe by reducing the P-gp or CYP450 enzyme's activity [65]. The inhibitory impact of QR and TQ on the activity of various cytochrome P450 (CYP) enzymes was investigated by Fawzy Elbarbry El et al., as these enzymes metabolize the majority of drugs available on the market. In-vitro experiments were conducted utilizing fluorescence-based high throughput assays and validated HPLC assays employing human c-DNA baculovirus produced enzymes. The following are the inhibitory effects of QR, TQ, and positive control inhibitors on the activity of the five CYP enzymes studied in Table 3 It was found from the research that both QR and TQ are moderate inhibitors of CYP2C19 (closely associate with metabolism of antineoplastic drugs like tamoxifen and cyclophosphamide), CYP2D6 (responsible for catalysing biotransformation of various drugs), and CYP3A4 (responsible for the metabolism of 50% of all pharmaceutical agents) and show insignificant effect on the activity of CYP1A2 and CYP2E1 [66]. In a study on Quercetin, by Mohos et al. the inhibitory effects of quercetin and its methyl, sulphate, and glucuronide metabolites were tested on cytochrome P450, CYP2C19, CYP3A4, and CYP2D6) enzymes. CypExpressTM Cytochrome P450 human kits were used for studying the in vitro inhibitory effects of QR and its conjugates on cytochrome enzymes. HPLC system was used to analyse substrates and the formed metabolites while their inhibitory effects were noticeably smaller than the positive controls, it was discovered that QR conjugates were stronger inhibitors of these enzymes than the parent drug. Quercetin and its metabolites, isorhamnetin and isorhamnetin-3-glucuronide, slightly inhibit CYP2C19 and 3A4 activity while having no effect on CYP2D6 activity. Additionally, it was shown that QR conjugates interacted with CYP enzymes and drug transporters. CYP2C19 and 3A4 were significantly inhibited by QR at doses of 5–20 µM (p < 0.01) (one- to four-fold concentration vs. The substrates). In CYP2C19 and CYP3A4 tests, metabolite production was shown to decrease by around 25–35% and 30% to 45%, respectively, in the presence of 30 µM QR concentration [4]. A brief overview of the studies conducted to check the action of QR on CYP enzymes is mentioned in Table 4.

**Table 3:** Inhibitory effects of QR, TQ, and positive control inhibitors on the activity of the five CYP enzymes

CYP enzyme	Substrate	Metabolite	Observation detection		Positive control inhibitor
CYP1A2	3-Cyano-7-ethoxycoumarin	3-Cyano-7-hydoxycoumarin	Fluorescence	Excitation: 410 Emission: 460	BZF
CYP2C19	3-Cyano-7-ethoxycoumarin	3-Cyano-7-hydoxycoumarin	Fluorescence	Excitation: 410 Emission: 460	TCP
CYP2D6	3-(2-[N, N-diethyl-N-methylamino] ethyl)-7-methoxy-4-methylcoumarin	3-(2-[N, N-diethyl-amino] ethyl)-7-hydoxy-4-methylcoumarin HCL	Fluorescence	Excitation: 390 Emission: 460	QND
CYP3A4	7-benzyloxy-trifluoromethylcoumarin	7-hydroxy-trifluoromethylcoumarin	Fluorescence	Excitation: 409 Emission: 530	KTZ
CYP2E1	Chlorzoxazone	6-Hydroxychlorzoxazone	HPLC–UV	287	ABT

**Table 4:** An overview of studies conducted to study the action of QR on CYP enzymes

Name of target enzyme	Type of study, method used	Mechanism of action	Level of inhibition	Type of cancer associated with the target enzyme	Dose	References
CYP2E1	In vitro, Assay Method	Hydroxylation of p-nitrophenol to 4-nitrocatechol	Moderate (46.7%)	Breast cancer	128 µm	[65]
CYP3A4	In vivo (rats), fluorometric high-throughput screening	O-Dealkylation of 7-benzyloxy-4-trifluoromethylcoumarin (BFC) to 7-hydroxy-4-trifluoromethylcoumarin (HFC)	51.02 ± 1.24%	Colon, breast, heptacullar cancer	1-25 µg/ml	[66]
CYP2D6	In vivo (rats), fluorometric high-throughput screening	Hydroxylation, demethylation, dealkylation	78.46 ± 1.32%	Breast cancer	1-25 µg/ml	[66]
CYP1A2	In vivo, humans, HPLC	phase I enzyme, activates carcinogens, interacts with aromatic amines and polycyclic aromatic hydrocarbons	45.85%	Lung cancer, colon cancer, breast cancer	500 mg capsule everyday	[67]
CYP2C9	In vivo, humans	Non-competitive inhibiton of 6-hydroxyflavone	16.73 ± 5.01	Colorectal cancer	Quercetin 500 mg capsule	[68, 69]

## Natural plant extracts as cytochrome inhibitors

### Liquorice

Liquorice (*Glycyrrhiza glabra*) has been reported to exhibit many medicinal properties [67]. It is included in a variety of herbal medications, nutraceuticals, and dietary supplements and has historically been used to treat a number of ailments [68]. According to reports, *Glycirrhiza. glabra* has chemo preventive efficacy against cancer via inducing nuclear factor (erythroid-derived 2)-like factor 2 (Nrf2) [69, 70]. It is seen that food and nutrition greatly influence the development of breast carcinogenesis. Breast cancer can be prevented by eating foods that change the activity of certain cytochrome P450 enzymes, including CYP1A1, CYP1A2, CYP1B1, CYP2B6, CYP3A4, CYP19A1, and CYP24A1. Which is why taking liquorice or its active constituents in the form of dietary supplements if being encouraged [71]. The capacity of 14 isolated liquorice chemicals to inhibit 9 cytochrome P450 enzymes was examined using a UHPLC-MS/MS cocktail assay. The drug metabolising enzyme CYP3A4 was observed to be moderately inhibited by an ethanolic extract, as were CYP2B6, CYP2C8, CYP2C9, and CYP2C19. A variety of CYP450 enzymes involved in drug metabolism were shown to be inhibited by extracts from the three liquorice species—*Glycyrrhiza glabra, Glycyrrhiza uralensis,* and *Glycyrrhiza inflata*—that are most often used in botanical dietary supplements. It was discovered that among the compounds unique to liquorice that inhibit these enzymes are glabridin, glycycoumarin, and most significantly licochalcone A. Since each species of liquorice has demonstrated a unique pattern of enzyme inhibition, different drug-botanical interactions may be expected. The liquorice raw materials used to make dietary supplements must thus be botanically recognised to the species level, with full disclosure of both the species identity and method of preparation/extraction on the product labels [72]. The objective of Tu et al. was to look into the interaction of glycyrrhizin and omeprazole, as well as the effects of glycyrrhizin on CYP2C19 and CYP3A4 activity in healthy Chinese male volunteers with various CYP2C19 genotypes. In a two-phase randomised crossover experiment, eighteen healthy volunteers (six CYP2C19*1/*1, five CYP2C19*1/*2, one CYP2C19*1/*3, five CYP2C19*2/*2, and one CYP2C19*2/*3) were included. All individuals received either a placebo or a 150 mg glycyrrhizin salt tablet twice daily for 14 days in each phase. By using high-performance liquid chromatography, the pharmacokinetics of omeprazole (20 mg orally on day 15) were evaluated for up to 12 h after delivery. In CYP2C19*1/*1, CYP2C19*1/*2 or *3, and CYP2C19*2/*2 or *3 subjects who received a single oral 20-mg dose of omeprazole (capsule) after 14 days of treatment with placebo (open circles) and glycyrrhizin, mean plasma concentration–time profiles of omeprazole (A), 5-hydroxyomeprazole (B), and omeprazole (closed circles) were observed. Glycyrrhizin causes omeprazole sulfoxidation catalysed by CYP3A4 and results in lower omeprazole plasma concentrations, but has no effect on omeprazole hydroxylation mediated by CYP2C19 [73]. A brief overview of the studies conducted to check the action of liquorice on CYP enzymes if mentioned in Table 5.

**Table 5:** An overview of studies conducted to study the action of liquorice on CYP enzymes

Name of compound	Name of enzyme/receptor involved	Experimental model used	Assay/analysis method	Possible mechanism	Dosage given	Results of the study	References
Glycyrrhizin	P-gp, CYP3A4	Caco-2 cell transwell model and rat liver microsome incubation system	LC–MS method	CYP450-mediated medication efflux, hastening the metabolism or efflux of co-administered toxic ingredients and by increasing their metabolism	20 mg/kg	Glycyrrhizin could decrease the system exposure of asiatic acid, possibly by inducing the activity of P-gp or CYP450 enzyme	[77]
Glycyrrhizin	CYP2C19 CYP3A4	18 healthy adult men who were not related from a total of 407 healthy Chinese volunteers	Two-phase crossover study design	Activation of Gal-hPXR, inducer of CYP3A4 and potential hPXR associate	Glycyrrhizin salt tablet 150 mg two times daily for 14 days	By boosting CYP3A4 activity in all three CYP2C19 genotypes of healthy Chinese male participants, glycyrrhizin accelerated the metabolism of omeprazole. After 14 days of exposure, glycyrrhizin had no effect on any subject's CYP2C19 activity	[76]
Glycyrrhizin + midazolam	CYP3A, CYP1A2, CYP2B1, CYP2E1 and CYP1A1	Sixteen healthy adult male subjects	Two-phase randomized crossover design	Needs to be examined	7.5 mg/kg	A 14-day therapy with glycyrrhizin led to a minimal induction of the CYP3A model substrate midazolam	[78]
Licochalcone A	CYP1A2, CYP2D6, CYP2E1, CYP2C19, CYP2C8, CYP2C9 and CYP3A4	Hunan liver micrzosomes	Single-point inactivation experiment	Licochalcone A. While acting as a competitive inhibitor for CYP3A4, demonstrated varied reversible inhibitory behaviours against CYP2C8, CYP2C9, CYP2C19, and CYP1A2 in the Human Liver Microsomes	50 microM	All of the examined CYPs in the 236 human liver microsomes were inhibited by licochalcone A. On the catalytic 237 activities of CYP1A2, CYP2C8, CYP2C9, CYP2C19, and CYP3A4, LCA showed strong inhibitory effects. In contrast, its inhibitory effects on the activities of CYP2D6 and CYP2E1 were 238 very moderate. At 10 µm licochalcone A, CYP1A2, 240, CYP2C8, CYP2C9, CYP2C19, and CYP3A4 had residual activity that, respectively, were 40.7%, 4.9%, 19.9%, 241 (43.6%), and 19.5% of the control	[79]

### Garlic

Anti-inflammatory, antioxidant, anticancer, and detoxifying properties are some of the few of the biological actions of this garlic and its constituents [74]. Garlic (*Allium sativum*) has the capacity to inhibit tumour growth due to a number of mechanisms, including the stimulation of detoxification enzymes, protection against oxidative stress, induction of cell apoptosis and cell cycle arrest, prevention of chromosomal damage, immune system stimulation, and suppression of nitrosamine bioactivation. On the other hand, cancer prevention may involve both genetic and epigenetic processes in addition to hereditary ones. This anticancer effect is known to be mediated by CYP450 [75]. The angiogenesis process is aided by the vascular endothelial growth factor receptor 2 (VEGFR-2). Inhibition of VEGFR-2 is predicted to effectively suppress the proliferation of breast cancer cells, particularly triple negative breast cancer. Allicin, found in solitary garlic cloves, acts as an antioxidant and anticarcinogen in the same way as N-Acetyl Cysteine does (NAC). In this study by Veterini Et al through an in-silico analysis, the Allicin molecule was assessed from solitary garlic as an angiogenic potential inhibitor of VEGFR-2 breast cancer [76]. The ability of garlic to significantly reduce 6-hydroxychlorzoxazone/chlorzoxazone serum ratios, indicated suppression of CYP2E1 (cytochrome P450 2E1) [77]. Cytochrome P450 phenotypic ratios for predicting herb-drug interactions in humans. Additionally, there was a significant inter-person variation in ritonavir pharmacokinetics following the administration of garlic, indicating that ritonavir and garlic together may function as an inducer and inhibitor of CYP3A4 (cytochrome P450 3A4) and P-gp (P-glycoprotein) activity. The effect of short-term administration of garlic supplements on single-dose ritonavir pharmacokinetics in healthy volunteers was studied which showed lowered cytotoxicity [78]. In a study conducted by Destiny M. Davenport it was revealed that there was a decrease in hepatic CYP2E1 protein by 45%, 25% and 47% on the administration of a single dose of 200 mg/kg of diallyl sulphide, diallyl disulphide, and allyl methyl sulphide respectively. These are organosulfur compounds of garlic that were gastrically intubated into rats and this inhibition sustained even after 1, 4 and 8 weeks after administration of these compounds respectively. Western blot and real time PCR were the methods used for detecting proteins in rat tissue samples and detecting effect on tumorous cell induced in rats respectively. Beta-actin was used as the internal control. In conclusion results from the studies concluded that organosulfur compounds present in garlic namely Diallyl sulphide and Allyl Methyl Sulphide may help in inhibiting colon cancer that is induced chemically, but prolonged dosing of diallyl Sulphide in higher concentrations might show signs of hepatotoxicity [79]. A brief overview of the studies conducted to check the action of garlic on CYP enzymes if mentioned in Table 6. In a study conducted by Manal et al. role of garlic in phase 1 reactions of biotransformation system in the liver of *Oreochromis niloticus* (species of fish) suffering from aflatoxicosis was researched. Hepatic histopathology and Real-time polymerase chain reaction was used to measure the level of CYP1A1 gene expression. Hepatosomatic indexes were also recorded. Aflatoxin B1 is responsible for lowering Hepato-Somatic Index Values in fish. It is a mycotoxin that causes carcinogenicity and hepatotoxicity in fish. It was found that administering low doses of garlic (10gm/kg) led to significant improvements in the Hepato-Somatic Index Values. AFB1-injected fish experienced more liver damage than control fish (no AFB1). When administered intravenously at a dose of 6 mg/kg body weight, aflatoxin B1 had a negative impact on HSI, liver histology, and CYP1A production. Garlic-eating *O. niloticus* aflatoxicated animals shown some evidence of protection. Through the suppression of CYP1A in the fish liver, garlic improved the evacuation and detoxification of aflatoxin B1 [80].

**Table 6:** An overview of studies conducted to study the action of garlic on CYP enzymes

Name of compound	Name of enzyme/receptor involved	Experimental model used	Assay/analysis method	Possible mechanism	Dosage given	Type of cancer associated; problems associated	Results of the study	References
Lupeol and betulin	CYP1A2, CYP2C9, CYP3A4	Rat liver microsomes	HPLC	–	20 mg/kg and 50 mg/kg for 14 days	–	Neither inhibition nor Induction	[81]
Diallyl sulfide	CYP2E1	Incubation of rat liver slices with acrylamide	liquid chromatography tandem mass spectrometric method	Acrylamide oxidation to Glycidamide is inhibted	100 µmol/litre	Ovarian, endometrial cancer, genotoxicity	Diallyl sulfide inhibits formation of free glycidamide from acrylamide	[82]
S-Allyl-l-cysteine sulfoxide	Epidermal growth receptor (EGFR)	In silico molecular docking studies and in vitro validation	Discovery studio software version 4.0, cell viability test, RT-PCR	cell cycle dysregulation and promotion of multiple signaling pathways of RAS-RAF-MAP kinase pathway and the PI3K-PTEN-Akt	EGFR 100 nmoles	Colorectal Cancer	High affinity towards EGFR, efficacious as a potent anticancer agent	[83]
Diallyl sulfone, diallyl sulfide	CYP2E1, CYP2B1	Liver microsomes from acetone-pre-treated male Sprague–Dawley rats	PNP hydroxylase assay	competitive inhibition of p-nitrophenol hydroxylase activity	188, 390, and 118 µM	Breast and prostate cancer	Inhibits metabolism of P-450 2E1 substrates by competitively inhibiting, inactivating P-450 2E1 via suicide-inhibitory action of DAS02	[84]
Allicin	CYP3A4, CYP3A5	Women with metastatic breast cancer	Blood sampling, Pharmacokinetic data analysis, genotype analysis	–	Docetaxel 30 mg/m^2^	Metastatic breast cancer	Decreases the clearance of docetaxel in patients carrying a CYP3A5*1A allele	[85]

## Clinical trials

We found three clinical trials which were relevant to our topic of interest. (Refer to Table 7) One of them investigated the potential activity of a novel quadrate combination therapy for treating early breast cancer comprising Mifepristone, Tamoxifen, Retinoic acid and Cannabidiol which is a selective CYP26 inhibitor. Cannabidiol, a major phyto cannabinoid, acted as a potent atypical inhibitor for CYP2D6 [86]. Another clinical trial though not extremely apt to our topic involves the idea of supressing a specific over expressed enzyme/hormone/gene for the treatment of a cancer. Hormone suppression therapy involves reduction or elimination of blood testosterone levels that may be detected. The tissue's testosterone concentrations are still high enough to activate androgen receptors, though. All cell lines that exhibit "androgen independence," or resistance to androgen-suppressive treatment, have overexpressed androgen receptors. Goserelin suppresses androgen production in turn helping in the therapy of prostate cancer [87]. This clinical experiment examined gene expression in men with metastatic prostate cancer who were being treated with cytochrome P450 17 alpha hydroxylase/17,20 lyase (CYP-17) inhibitors. Using tissue, blood, and urine samples from patients receiving CYP-17 inhibition medication, researchers were able to better understand DNA modifications that take place as well as find cancer-related biomarkers. It also assisted medical professionals in understanding how effectively individuals respond to therapy [88].

**Table 7:** Clinical trials related to CYP enzyme inhibition and cancer therapy

Sr. No.	NCT No.	Study description	Disease/condition	Intervention/treatment	Phase (status)	Study type	References
1.	NCT05016349	Investigating the potential role of a novel quadrate combination therapy mifepristone (Antiprogestrone), Tamoxifen, retinoic acid and cannabidiol (selective Cyp 26 inhibitor) for treating early breast cancer	Breast cancer: female	Drug: retinoic acid Vesanoid Drug: 13-cis retinoic acid plus tocopherol 13-Cis retinoic acid (50 mg/m^2^/day) Tocopherol (800 mg/day) Drug: Mifepristone Mifepristone Drug: cannabidiol Liquid Other name: epidiolex Drug: 9 cis retinoic acid Drug: Tamoxifen Drug: standard therapy Patients will receive the approved standard therapy tamoxifen	Phase 3	Intervention	[86]
2.	NCT00298155	Maximal suppression of the androgen axis in clinically localized prostate cancer (TAPS)	Cancer Prostate neoplasms	Drug: goserelin with dutasteride Bicalutamide Drug: goserelin with bicalutamide and dutasteride Drug: goserelin with bicalutamide and dutasteride and ketoconazole	Phase 2	Intervention	[87]
3.	NCT01953640	Gene expression in patients with metastatic prostate cancer receiving CYP-17 inhibition therapy (PROMOTE)	Hormone-resistant prostate cancer Metastatic prostate carcinoma Prostate adenocarcinoma Recurrent prostate carcinoma	Other: laboratory biomarker analysis Other: quality-of-life assessment	Active, not recruiting	Observational	[88]

## Conclusion and future prospects

The present literature review led to a conclusion that thymoquinone, quercetin, garlic extract and liquorice extract have proven themselves to be inhibitors of the different enzymes within the CYP family of enzymes. The potential of these natural cytochrome p450 inhibitors should be used to its fullest by developing formulations of anticancer drugs in synergy with natural cytochrome p450 inhibitors so as to increase their anti-neoplastic activity. Herb drug interactions with Cytochrome enzymes need to be explored as almost every drug get metabolised by them. Liposome, micelles, nanoparticles, and other novel drug delivery systems have the potential to be utilised for the establishment of Hepatocellular targeted drug delivery systems. In addition, Quantum mechanical calculations, computer-aided drug design, and in silico protein-drug interactions investigations will make it possible to understand how P450 interacts with herbal ingredients. Consequently, speculations about unidentified aspects of human P450 may be investigated, and predictions can be made about the outcome of an interaction between herbal elements within a particular P450. The interactions, pertaining to the processes on such binding, might be analysed using computational methods, assisting in the prediction of novel active site conformations. Lead molecules and herbal components can be oriented by adding some of the active site's calculated contact residues to the model.

## Data Availability

The data that support the findings of this study are available from the corresponding author, [Sankha Bhattacharya], upon reasonable request.

## Abbreviations

CYP: Cytochrome P450
BZF: 7,8 Benzoflavones
TCP: Tranylcypromine
QND: Quinidine
KTZ: Ketoconazole
ABT: Aminobenztriazole
HPLC–UV: High-performance liquid chromatography–ultraviolet
QR: Quercetin
TQ: Thymoquinone
